# Determining the MRI Examination Experience: The Relationship Between Waiting Times, Information Provision, and Patient Anxiety

**DOI:** 10.3390/healthcare14172716

**Published:** 2026-08-25

**Authors:** Ádám Román, Ida Ercsey, Helga Judit Feith

**Affiliations:** 1 Health Sciences Division, Semmelweis University Doctoral College, 1085 Budapest, Hungary; roman.adam@phd.semmelweis.hu; 2 Kautz Gyula Faculty of Economics, Department of Marketing and Management, Széchenyi István University, 9026 Győr, Hungary; ercsey@sze.hu; 3 Department of Social Sciences, Faculty of Health Sciences, Semmelweis University, 1085 Budapest, Hungary

**Keywords:** magnetic resonance imaging (MRI), patient pathway, waiting time, patient experience, MRI-related anxiety, imaging, preventive imaging, patient satisfaction

## Abstract

**Background:** Performance evaluation in MRI diagnostics traditionally focuses on operational metrics (e.g., waiting times), neglecting patient experience, which fundamentally determines patients’ future healthcare compliance. The aim of our study was to identify the key factors determining patient satisfaction, particularly with regard to human interactions and the psychological impacts of the examination environment. **Methods:** A cross-sectional questionnaire survey was conducted at eight public diagnostic centers in Hungary (N = 742). Predictors of overall satisfaction were analyzed using multivariate linear regression (IBM SPSS 27.0), examining the effects of structural (waiting list), environmental (noise, isolation, and hygiene), and interpersonal (professional competence and information provision) variables (*p* < 0.05; 95%CI). **Results:** The model explained 48.8% of the variance in patient satisfaction. The most significant positive predictor was healthcare staff competence (β = 0.349; *p* < 0.001), followed by confidence in the effectiveness of the examination (β = 0.208) and hygiene (β = 0.128). The distressing effect of loneliness (isolation) was identified as a key negative factor (β = −0.131; *p* < 0.001). In contrast, waiting time and acoustic load (noise) were not significant confounding factors in the final model. **Conclusions:** Our findings highlight that, according to patients’ subjective experiences, the quality of radiological care is determined by human factors and clinical trust rather than operational speed. To improve patient satisfaction, emphasis should be placed not only on technical advancements but also on enhancing staff communication competencies and mitigating the experience of isolation during examinations.

## 1. Introduction

Magnetic resonance imaging (MRI) has become one of the fundamental tools of diagnostic care in recent decades [1,2]. Consistent with international trends, the number of MRI examinations has increased significantly, which, on the one hand, contributes to improved diagnostic accuracy and, on the other hand, places increased pressure on the healthcare system [3]. This growing demand presents a particular challenge regarding waiting times and resource allocation [4,5]. Consequently, MRI services are often evaluated using structural performance indicators such as access, waiting lists, and capacity utilization [6]. As a result, access to MRI has become an issue of not only organizational relevance but also broader health policy significance [7,8].

Optimizing structural access has been the focus of numerous studies. Streit et al. demonstrated through a detailed analysis of MRI workflows that scanner capacity is often underutilized and that the organization of operational processes—such as patient registration, preparation, and exam scheduling—significantly influences the efficient use of resources [9]. Silva-Aravena et al. showed, using a digital twin and reinforcement learning approach, that optimized MRI scheduling can significantly improve equipment utilization while reducing waiting times [10]. These findings highlight that access to MRI can be enhanced through organizational and technological interventions; however, structural optimization alone does not necessarily capture the full spectrum of service quality [7,11].

These structural challenges are particularly pronounced in centralized, publicly funded systems such as Hungary. In the Hungarian public healthcare sector, high-capital diagnostic equipment—such as MRI scanners—is heavily concentrated in county-level hospitals and regional centers [12]. Due to public capacity limits and strict budget caps, patient pathways are often characterized by prolonged structural waiting times, averaging over 50 days for elective scans [13]. Within this institutional framework, patients frequently perceive administrative delays as systemic baseline conditions of public care. Consequently, their evaluation of service quality relies heavily on their immediate, in-person interactions with radiological staff once care is delivered [14].

Patient satisfaction plays a central role in assessing healthcare quality. The literature clearly supports the notion that a positive patient experience and satisfaction are associated with clinical safety, treatment adherence, and the effectiveness of care [15,16]. According to the WHO, patient satisfaction is a fundamental indicator of healthcare quality, determining the quality of service as experienced by patients and confidence in the healthcare system [17]. In radiology services, where the diagnostic significance of examinations is paramount, it is particularly important to identify factors that influence patient satisfaction during examinations [18,19,20].

The experience of an MRI examination is a complex phenomenon influenced by physical, psychological, and communication factors [21,22]. The enclosed space, acoustic noise, and the feeling of loss of control often trigger anxiety or a claustrophobic reaction [23,24]. Severe anxiety can lead to premature termination of the examination or patient movement during the procedure, compromising image quality and reducing diagnostic value. In clinical settings, the rate of examination interruptions attributable to claustrophobia can reach 1% [23]. Patient perceptions of the examination are further influenced by diagnostic uncertainty, indication for the examination, and concerns regarding the prognosis [25]. Effective communication, adequate pre-examination information, and the establishment of good rapport have been shown to reduce anxiety and improve patient cooperation during the examination [26,27].

Al Shanbari et al. conducted an empirical study of anxiety associated with MRI examinations involving 465 patients and found that more than half of the participants experienced moderate anxiety during the examination; however, female patients reported significantly higher levels of anxiety. Their findings highlight that the psychological dimensions of MRI examinations are clinically relevant and may directly influence the assessment of examination quality [28].

Alghamdi examined patient satisfaction with radiology services across ten hospitals, with the majority of patients reporting high satisfaction; however, waiting times were associated with lower satisfaction [18]. Another study evaluating service quality [29] emphasized that personal communication, detailed information, and emotional support are key factors in creating a positive MRI experience for patients. Experiences gained during the examination influence not only perceptions of the procedure itself but also the overall evaluation of service quality [30].

Overall, it can be concluded that the evaluation of MRI services is based on two interrelated dimensions: on the one hand, structural access and waiting times [31]; on the other hand, the quality of care experienced during the examination and patient satisfaction [32,33]. Although these areas are well documented individually, few studies are available that analyze these two factors together within an integrated framework. For a comprehensive evaluation of the quality of diagnostic services, it is necessary to explore how access indicators and examination experience are related and how they contribute to the quality of service as perceived by patients.

To address this gap within a rigorous theoretical framework, our analytical findings are conceptualized through Donabedian’s Structure–Process–Outcome (SPO) quality assessment model [34]. While our stepwise regression model identifies the most parsimonious statistical predictors based on empirical variance explanation, the variables naturally align across Donabedian’s three dimensions:

1. Structure: Operational access and physical surroundings (waiting time, hygiene, and acoustic noise).
2. Process: Interpersonal care delivery and psychological management (staff competence, information provision, and spatial isolation).
3. Outcome: Evaluative cognitive perceptions (confidence in diagnostic effectiveness and safety) and overall satisfaction.

This framework allows us to test whether process-level human interactions can buffer structural access deficits in public healthcare. To the best of our knowledge, this is the first time a comprehensive, complex study of this kind has been conducted, particularly in Hungary.

## 2. Materials and Methods

**Figure 1:**
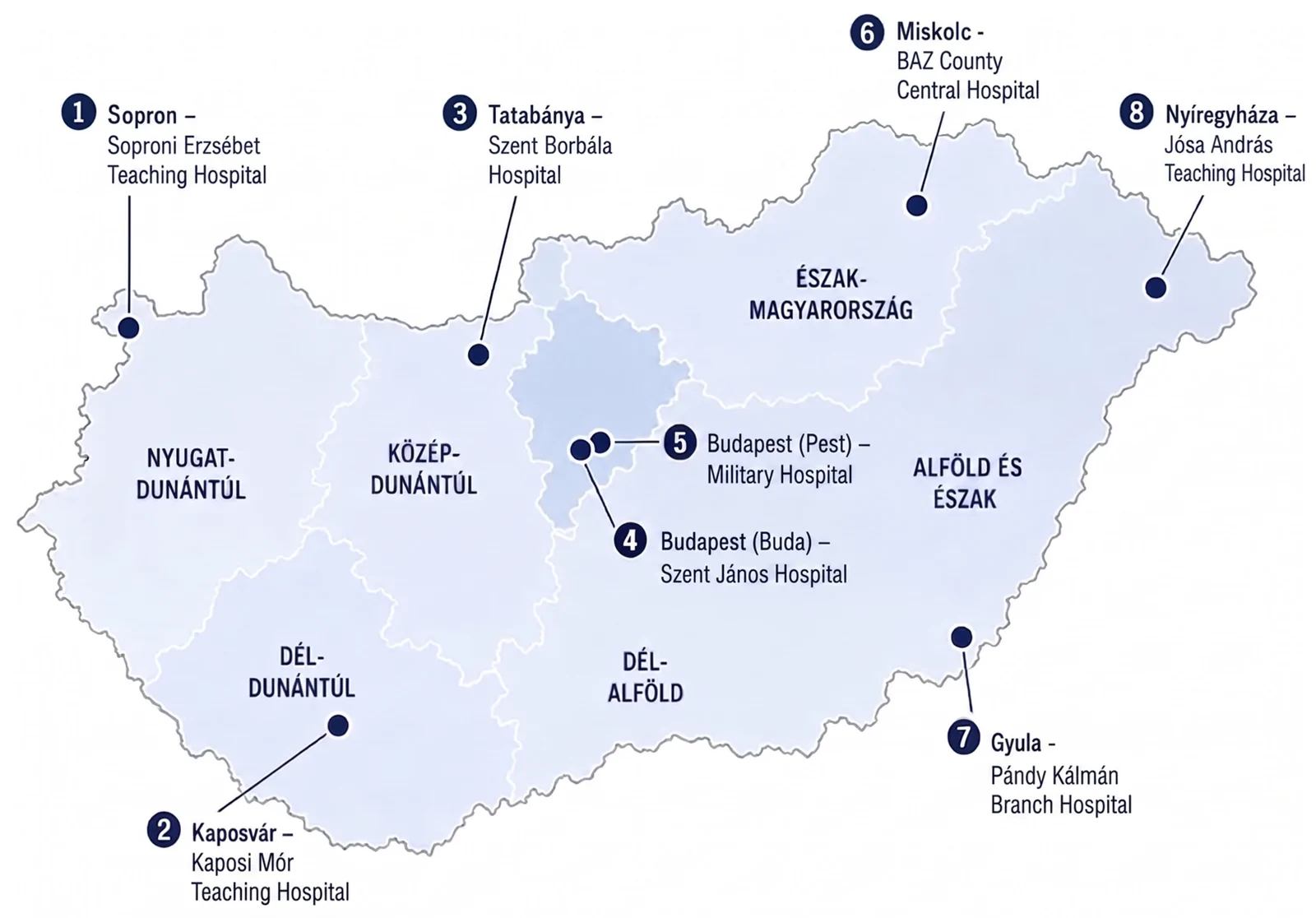
Institutions in cities with county rights included in the study.

Before sampling began, the study received official approval. The ethical clearance number certifying the ethical basis of the research is BM/26297-1/2023. Target institutions for the study were hospitals in Hungary equipped with MRI machines. Sampling was conducted through purposive selection, taking into account the institutions’ geographical locations and the availability of MRI services. During data collection, sampling was conducted across all NUTS 2 regions in Hungary (Figure 1). The selection of institutions within each region was based on the 2023 hospital bed count report published by the National Health Insurance Fund of Hungary [35]. University clinical centers within the NUTS 2 region were excluded because of their extremely large bed capacity, unique regional location, and level of progressiveness, which would have significantly distorted the comparison. As a result, the study better captured the institutional differences within the classic public hospital structure.

The study included one state-funded hospital located in a city with county rights in each NUTS 2 region, as specialized outpatient care capacities in Hungary are concentrated in these types of settlements. To ensure comprehensive geographical representation across all NUTS 2 regions, a pragmatic purposive sampling strategy was employed. While priority was given to the largest county-level non-university healthcare centers, secondary regional hubs—specifically the Erzsébet Teaching Hospital and Rehabilitation Institute in Sopron and the Szent Borbála Hospital of Komárom-Esztergom County in Tatabánya—were selected based on their high patient volume, key regional coverage, and institutional feasibility for multi-center data collection.

In the case of the Budapest and Pest NUTS 2 region (formerly the Central Hungary region), two institutions were included in the analysis. A key consideration was ensuring representation of both a Buda-based and a Pest-based institution, given the differences in the socioeconomic characteristics of the patient populations [35]. On the Pest side, the Military Hospital currently has the largest number of beds among non-clinical-level institutions, while on the Buda side, János Hospital has the largest number of inpatient beds.

In total, the study was conducted at eight healthcare facilities.

Data were collected using a self-administered, paper-based questionnaire completed on-site by patients immediately after their MRI examination. The survey instrument was developed through a structured, multi-stage process. Questionnaire items were adapted from established radiological quality frameworks and validated patient satisfaction scales. To ensure content and face validity, the preliminary item pool was evaluated and refined by a panel of senior radiologists, chief radiographers, and healthcare quality specialists. Before full administration, a pilot pre-test (n = 25) was conducted to confirm item readability, clarity, and completion time, leading to minor phrasing refinements. During the study period in 2025, a total of 742 adult patients (aged ≥18) voluntarily participated in the survey at one of the eight designated institutions. The questionnaire was completed anonymously following written informed consent. Inclusion criteria for the study sample were undergoing an MRI examination at a participating institution; being of legal age and legally competent; being able to complete the questionnaire independently; and completing and signing the consent form. Exclusion criteria were not meeting the inclusion criteria and incomplete questionnaire responses (defined as <80% completion). A total of 756 responses were received, of which 14 were excluded for not meeting the minimum completion threshold.

Descriptive statistical analyses were also conducted during the quantitative research. In this context, mean, standard deviation, median, and minimum and maximum values were calculated for continuous variables, while frequency measures were used to present categorical variables. Multivariate regression analysis was performed to identify factors influencing overall satisfaction with the MRI examination. Variables representing organizational, communication, environmental, personal, and perceptual dimensions of the care process were included in the model. Factors examined included waiting lists, the quality of information provided, the distracting effect of the high-pitched noise during the scan, and the sense of isolation during the procedure. Additional considerations involved personal aspects of the MRI examination, such as staff competence and hygiene. In the present study, staff competence referred to patients’ overall subjective perception of the competence demonstrated by healthcare professionals during the MRI examination. This measure should not be interpreted as an objective assessment of technical or clinical competence. Rather, patients’ evaluations are likely to reflect a global impression based on observable aspects of care, including the clarity of instructions, the confidence demonstrated by staff, responsiveness to patients’ needs, the quality of communication, and the perceived safety and organization of the examination process. Perceptions of the overall quality of the MRI examination were also assessed, particularly regarding effectiveness and safety. The stepwise method was applied to conduct the hierarchical multiple regression analysis. The variable entered first has the highest partial correlation in absolute value. In addition, the stepwise method ensures that only predictive variables that significantly explain the variance of the outcome variable remain in the regression model. The structure of the questionnaire was evaluated using factor analysis. Internal consistency reliability of the study instrument was assessed using Cronbach’s alpha coefficient. The key assumptions of linear regression, including the normality of regression residuals, homoscedasticity, and the absence of multicollinearity, were assessed using standard diagnostic procedures. To assess the robustness of the findings, we conducted a sensitivity analysis using an alternative model specification. The diagnostic results indicated that these assumptions were adequately satisfied, supporting the appropriateness of the fitted regression models.

Data recording and statistical analyses were performed using IBM SPSS Statistics version 27.0. Results were considered statistically significant at a 95% confidence interval (*p* < 0.05).

## 3. Results

The satisfaction dimension was assessed using six variables, which were rated on a 1–4 Likert scale.

Factor analysis was performed to confirm the structure of the questionnaire. The results of the exploratory factor analysis demonstrated that the data were suitable for factor analysis (KMO = 0.759), exceeding the commonly accepted threshold of 0.60 reported in the literature. The extracted factors jointly explained 63.7% of the total variance, which can be considered a satisfactory result. The first factor comprises five variables and represents the Personal Factors—Trust dimension. The second factor consists of three variables reflecting the psychological burden experienced by patients during MRI examination. For the first factor, factor loadings ranged from 0.642 to 0.848, indicating moderate-to-strong associations between the variables and the underlying factor. As all factor loadings exceeded 0.60, it can be concluded that the items belonging to the first factor provide a good representation of the underlying construct. The factor loadings for the second factor ranged from 0.780 to 0.888, which are considered particularly high. This indicates that the variables associated with the second factor are very strongly related to the common latent dimension, suggesting that this factor has a well-defined and stable structure. Overall, the results indicate that the two factors are clearly distinguishable, and the high factor loadings of their respective variables support the construct validity of the measurement instrument.

The reliability of the scales was assessed using Cronbach’s alpha coefficient. The results indicated that the Staff Trust scale (F1) demonstrated good internal consistency (Cronbach’s α = 0.812), exceeding the commonly accepted threshold of 0.70 reported in the literature. Therefore, the scale can be considered reliable. Similarly, the Psychological Burden scale (F2) exhibited satisfactory internal consistency, as evidenced by a Cronbach’s alpha coefficient of 0.797. This value also exceeds the generally accepted threshold of 0.70, indicating that the scale possesses adequate reliability.

Overall patient evaluations of the MRI examination were favorable. The average overall satisfaction score was 3.76 ± 0.473. The most highly rated aspects were staff competence (3.87 ± 0.406), hygiene (3.77 ± 0.474), and confidence in the effectiveness (3.66 ± 0.517) and safety (3.67 ± 0.537) of the examination. The disruptive effect of the beeping sound received a lower rating (2.10 ± 0.944), whereas the perceived disruption of being alone was minimal (1.64 ± 0.845). Access to MRI scanning varied considerably, with a mean waiting time of 51.71 ± 53.7 days and a median of 31 days. Staff kindness was excluded from the regression analysis due to its strong correlation with competence (r = 0.769; *p* < 0.001). The number of missing responses varied across variables, with the highest rates observed for waiting time (n = 115) and overall satisfaction (n = 43) (Table 1).

The initial database contained 742 cases; however, due to missing data and the application of listwise exclusion, the final sample for the analyses was reduced to 581 individuals. Listwise missing-data handling was justified because the proportion of missing data was relatively low (3–6%) for each variable. This method did not result in any significant loss of information, while ensuring that all analyses were carried out on the same sample, thereby avoiding bias. However, for one variable in the regression analysis—the waiting list—the number of missing responses exceeded 10 percent (15.5%). The final sample size can be considered sufficient for carrying out the chosen statistical procedure; however, it should be borne in mind that the reduction in the number of observations due to missing data may affect the accuracy and generalizability of the results. We considered it important to include this among the limitations of the research.

Overall patient satisfaction and factors influencing their evaluation of the MRI examination.

Multivariate regression analysis was used to examine factors influencing overall satisfaction with the MRI examination, which included the following: waiting list, information provided, the distracting effect of the beeping sound, being alone during the examination, personal factors related to the MRI examination (staff competence and hygiene), and perceptions related to the quality of the MRI examination (effectiveness and safety of the MRI examination).

In the first model, staff competence has a statistically significant effect (β = 0.591) on satisfaction with the MRI examination. As satisfaction with staff competence increases, overall satisfaction also increases. The model is significant (F = 310.96, *p* < 0.001).

In the second model, predictors of staff competence and confidence in the effectiveness of the examination account for 43.4% of the variance in satisfaction (R^2^ = 0.434). The predictor variable professional competence has a significant positive influence on overall satisfaction (β = 0.482; t = 14.430; *p* < 0.001). In the second model, the inclusion of the variable confidence is also significantly associated with satisfaction (β = 0.311; t = 9.316; *p* < 0.001) and accounts for 8.5% of the variance in satisfaction with the MRI examination.

The perception variable in the third model of the regression analysis accounted for 2.7% of the variance in satisfaction with the MRI examination. The disturbance effect of being alone significantly influences overall satisfaction; the effect of the predictor variable is negative (β = −0.166; t = −5.336; *p* < 0.001).

Overall satisfaction with hygiene has a positive effect on satisfaction with the MRI examination (β = 0.160; t = 4.132; *p* < 0.001). This predictor variable plays a relatively minor role in assessing satisfaction, increasing the explanatory power of the model by approximately 1.5%.

In the fifth model, the inclusion of the variable confidence in the safety of the examination is also significantly associated with satisfaction (β = 0.101; t = 3.007; *p* = 0.011).

In the fifth model, the inclusion of the new variable (trust in the safety of the examination) contributed only marginally to the explanation of satisfaction (ΔR^2^ = 0.006).

Finally, the sixth model shows that the quality of information provided regarding the MRI examination also influences overall satisfaction (β = 0.082; t = 2.443; *p* = 0.015). The explanatory power changed to a similar extent in the sixth model (ΔR^2^ = 0.005) due to the inclusion of the evaluation of information quality. The standardized regression coefficients indicate that, among the confidence-related factors, confidence in the effectiveness of the examination (β = 0.208) has a greater impact on overall satisfaction than the perception of safety (β = 0.100). In terms of personal factors, the competence of healthcare professionals (β = 0.349) is more significant for overall satisfaction than satisfaction with hygiene (β = 0.128; t = 3.340; *p* = 0.001).

In the present study, the confounding effects of two variables—waiting list and the beeping sound—were excluded (t = −0.216, *p* = 0.829 and t = 1.476, *p* = 0.141).

During the construction of the hierarchical regression model, we assessed differences in patient satisfaction between institutions and regions. However, in the multivariate regression analysis, geographical differences did not directly influence changes in satisfaction. As a follow-up to this research, it may be interesting to investigate whether patient evaluations at specific institutions or in specific counties have a moderating effect on overall satisfaction with MRI examinations. Furthermore, during the statistical analysis, we used bivariate statistical methods to examine the associations between potential confounding variables and satisfaction with the MRI examination. For several variables, including MRI scan duration, type of symptoms, age, gender, and educational attainment, only minor differences in mean satisfaction scores were observed between groups, and these differences were not statistically significant. Among the variables examined, only previous experience was significantly associated with satisfaction. Patients with previous negative experience reported significantly lower satisfaction (3.59) than those without such an experience (3.77). This variable was subsequently included in the multivariate regression analysis; however, previous negative experience did not make a significant contribution to explaining satisfaction with the MRI examination.

In summary, 48.8% of the variance in satisfaction with the MRI examination is explained by the six predictor variables included in the model, while the remaining variance (over 50%) is accounted for by other factors not included in the model. A key determinant of satisfaction with the MRI examination was, on the one hand, the assessment of personal factors and staff competence, and on the other hand, patient perceptions of confidence in the effectiveness of the examination. In contrast, the feeling of isolation experienced during the MRI examination had a negative impact on satisfaction. Hygiene, perceived safety, and information provision had a comparatively smaller influence on overall satisfaction.

Key results of the hierarchical regression analysis are presented in Table 2.

Based on the regression model assumptions, the standardized residuals showed a near-normal distribution, as confirmed by both the histogram and the Q–Q plot. The Durbin–Watson statistic was 2.135, indicating that the errors were independent. The scatter plot of the standardized residuals and the standardized estimated values did not indicate any heteroscedasticity.

When examining multicollinearity, we analyzed the tolerance and VIF values. The tolerance values exceeded the 0.10 threshold for all variables, while the VIF values did not exceed the limit of 5. On this basis, it can be concluded that there is no significant multicollinearity in the model, and the regression coefficients can therefore be interpreted reliably.

Table 3 shows the VIF and tolerance values for the variables in the hierarchical regression analysis.

The final model explained almost half of the variance in patient satisfaction, indicating that the predictors included accounted for a substantial proportion of differences in satisfaction scores. However, approximately half of the variance remained unexplained, suggesting that other factors not included in the model also influence patient satisfaction. These may include unmeasured patient characteristics and factors relating to healthcare providers. For example, a patient’s expectations regarding the service and their previous experiences may also play a significant role in determining their level of satisfaction. Furthermore, in the present study, several important factors (such as the type of complaint, waiting list, duration of the MRI scan, and the patients’ socio-demographic characteristics) were included in the assessment of satisfaction; however, the bivariate and multivariate statistical analyses did not confirm the hypothesized effects. It was assumed that perceptions of the length of the waiting list would influence overall satisfaction; however, the significance test did not confirm this. This may be due to the relatively high level of missing data for this variable. A more detailed consideration of these factors highlights the complexity of patient satisfaction and underlines the need for future research to incorporate a wider range of individual and organizational factors in order to improve the explanatory power of predictive models.

To evaluate the robustness of the findings, a sensitivity analysis was performed using an alternative model specification to determine whether the results of the hierarchical regression model were preserved. The sensitivity analysis demonstrated that the regression coefficients were generally stable across model specifications. For three predictor variables—*competence of healthcare professionals*, *disruptive effects of being alone*, and *information provided*—the regression coefficients changed by approximately 10% following the application of the alternative specification. In contrast, the regression coefficients for effectiveness, safety, and hygiene remained unchanged. Furthermore, the pattern of statistical significance was fully retained, with the same three predictors remaining non-significant in the alternative model. The explanatory power of the model showed only a negligible increase, with the coefficient of determination changing from R^2^ = 0.488 to R^2^ = 0.490 (ΔR^2^ = 0.002). Taken together, these findings support the robustness of the hierarchical regression model, indicating that the alternative model specification did not produce any substantively meaningful changes in either the estimated regression coefficients or the overall model fit.

## 4. Discussion

**Figure 2:**
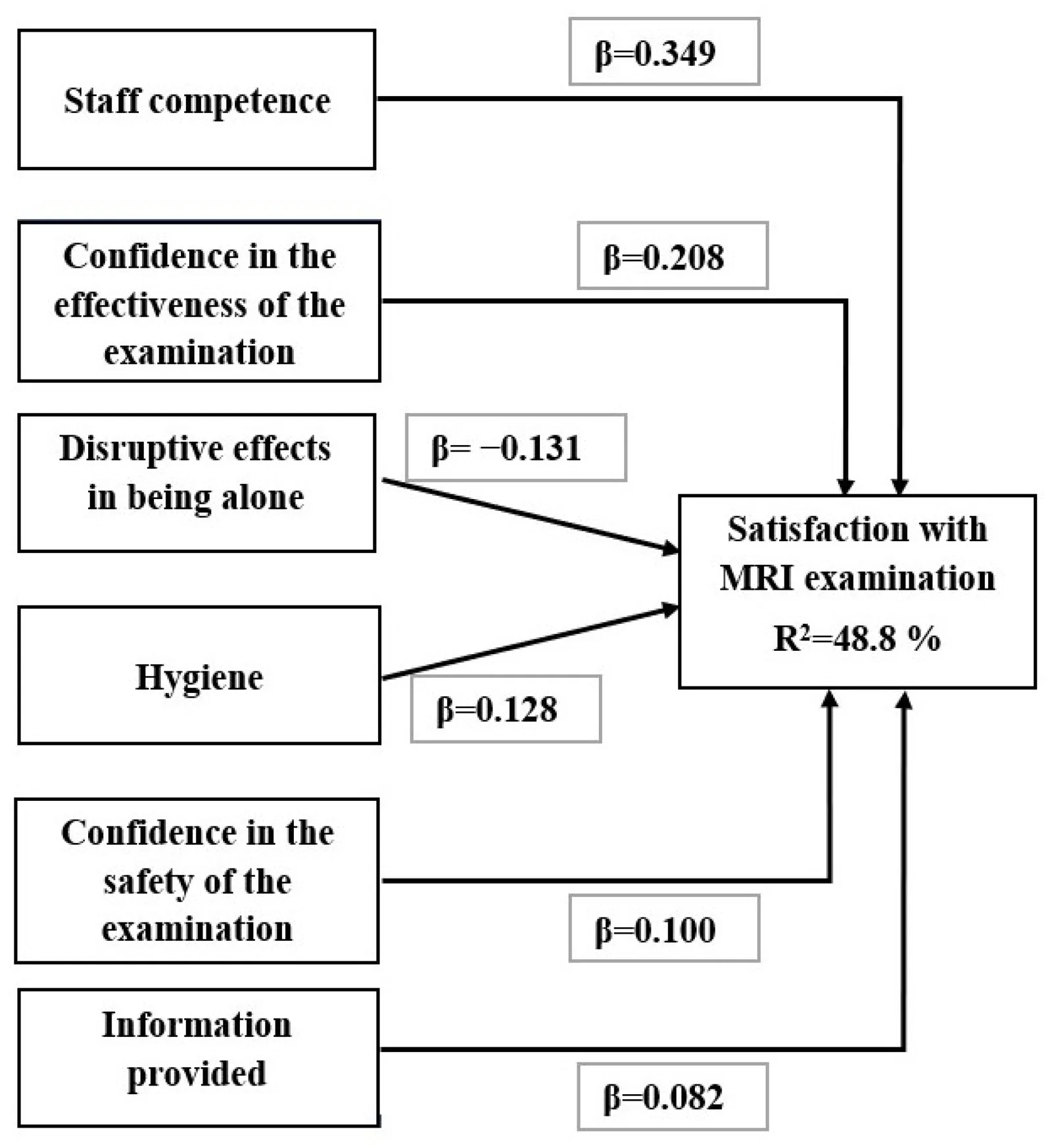
Significant predictors of satisfaction with the MRI examination in the final regression model.

The aim of our study was to use a multivariate regression model to identify the most important factors determining patient satisfaction with MRI examinations. Our results are consistent with international findings, which indicate that experience with MRI is shaped by both technological and procedure-related factors, such as examination environment, noise levels, and waiting times, as well as by aspects of human interaction, particularly staff communication, patient-centered information provision, and establishment of confidence in the examination [21,27,36]. Previous studies in radiology and MRI settings indicate that staff behavior and communication, along with waiting time, are among the most important determinants of patient experience, while targeted, patient-centered communication has also been shown to reduce MRI-related anxiety [19,31]. The 48.8% of variance explained by the final model (Figure 2) can therefore be interpreted as indicating strong explanatory power within the context of regression models examining subjective, psychological, and attitudinal outcomes. This also corresponds to a substantial effect size according to Cohen’s traditional effect size logic.

The role of healthcare professionals

The most important finding of our study is that the competence of healthcare professionals proved to be the strongest positive predictor. In fact, this variable was already dominant in the first model, and its effect remained significant even after the inclusion of other variables. Our results show that patients form their professional assessment of the staff not solely based on technical competence but also on communication skills, empathy, and professional demeanor [37,38]. During MRI examinations, which are technologically complex and often anxiety-inducing for patients, staff competence can serve as a particularly important indicator of quality, as it contributes to the predictability of the procedure, the patient’s sense of security, and the development of confidence in the examination process [38,39]. Through the expertise of healthcare professionals, patients perceive not only technical competence but also empathetic attention and a confident, supportive presence, which significantly shape the overall experience of the examination [37].

Confidence in the effectiveness of the examination and sense of safety

An interesting dichotomy emerges when analyzing confidence-related factors. Our data show that confidence in the effectiveness of the examination is a stronger determinant of satisfaction than the perceived sense of safety during the procedure. This shows that patient attitudes toward MRI examinations are fundamentally shaped by their hope and confidence in the diagnostic value: the primary goal for patients is to obtain an accurate diagnosis, and for this purpose, they appear willing to tolerate the discomfort associated with the examination. At the same time, appropriate communication and advance information remain essential, as they reduce patient anxiety, enhance overall satisfaction, and help patients perceive the examination not only as safe but also as diagnostically meaningful [22,27]. For clinical practice, these findings imply that patient information should not focus solely on risk reduction; it is at least equally important to clearly communicate the purpose, expected benefits, and diagnostic significance of the examination for patients [22].

Examination-related anxiety

The only consistently negative predictor in the model was the disruptive effect of being alone. This finding is consistent with the international literature on MRI, which indicates that the enclosed space of the examination room, the requirement to remain motionless, and the associated sense of loss of control cause significant stress for patients [39,40]. Qualitative studies similarly highlight that supportive interaction with the radiographer, trust-based communication, and adequate pre-examination preparation play key roles in managing fear, discomfort, and perceived loss of self-control, whereas the absence of these factors negatively affects the examination experience [39]. Our findings clearly demonstrate that the feeling of confinement alone can reduce patient satisfaction. Although hygiene and quality of information provided are important determinants of patient experience, this conclusion is consistent with radiology satisfaction surveys in which cleanliness, comfort, access to information, and service organization are repeatedly identified as key factors; however, the literature shows that these positive factors do not necessarily fully offset the negative psychological burden associated with MRI examination conditions [21].

Surprisingly non-significant factors: waiting list and noise

One of the most interesting findings of the study is that, contrary to our initial expectations, the length of the waiting list and the high-pitched noise typical of MRI scans did not remain significant factors in the final model. Previous studies in the literature suggest that waiting time can influence patient satisfaction; however, this effect is often mediated more by patients’ perceived or subjectively experienced waiting time than by the actual duration of the wait [28]. Accordingly, among patients who have already undergone the examination, earlier administrative delays may recede into the background, particularly if the experience during the procedure itself is positive [16]. Similarly, MRI noise is widely recognized in the literature as an important source of physical discomfort. Nevertheless, reviews on patient experience indicate that overall satisfaction is primarily shaped by personal interactions, including the quality of communication, a sense of control, and confidence in the diagnostic value of the examination [21,41]. While structural variables such as waiting time and acoustic noise failed to reach statistical significance in the final model, this finding does not imply their irrelevance, but rather illustrates how their predictive effect is mediated by care delivery [42].

Framed through Donabedian’s Structure–Process–Outcome model [34], the empirical stepwise selection provides key theoretical insights. High process quality—namely staff competence (β = 0.349)—exerts a protective buffering effect against structural friction such as waiting times and environmental noise [42]. In public healthcare settings, once patients enter the examination environment, humanized process quality supersedes structural access deficits in driving overall satisfaction.

Contextualizing this dynamic within Central and Eastern European public healthcare systems suggests a unique psychological prioritization [43]. Where resource constraints and prolonged waiting lists are common, patient expectations regarding operational speed and comfort are often calibrated downward. Consequently, structural delays do not trigger proportional dissatisfaction if care delivery is safe and empathetic [43]. This contrasts with high-resource or private settings in Western Europe and North America—where access times dominate satisfaction scores [14]—and helps explain how process quality effectively offsets structural deficits in state-funded diagnostic care.

Our findings suggest that patients tend to regard technological characteristics and administrative constraints partly as given conditions, whereas factors directly influencing the perceived quality and usefulness of the examination have a greater impact on their assessment of satisfaction.

Strengths and Limitations

In addition to its large sample size and comprehensive nationwide coverage, a key strength of this study lies in its multidimensional assessment of patient satisfaction with MRI examinations, incorporating technological, organizational, psychological, and interpersonal interaction factors simultaneously. The use of a multivariate regression model enabled the identification of the relative contribution of individual predictors and highlighted that staff competence, confidence in the effectiveness of the examination, and the experience of isolation are key determinants of patient experience. The practical value of the study is further reinforced by the direct applicability of its findings to radiology quality improvement initiatives, particularly in the areas of patient-centered communication, pre-examination information provision, and examination support [44].

The most significant limitation of this study is its cross-sectional design, which precludes causal interpretation of the observed associations. Furthermore, the pragmatic purposive sampling strategy across eight public hospitals—without randomized institutional selection—introduces potential selection bias. Although the sample offers broad regional representation of the Hungarian public sector, it specifically reflects non-university, state-funded hospital environments. Consequently, caution is required when generalizing these findings to private diagnostic imaging centers or university teaching hospitals, where resource availability, operational workflows, and patient expectations may substantially differ.

A further limitation of this study relates to the measurement instrument. Due to the unique institutional characteristics of the Hungarian public healthcare context and the lack of previously validated national surveys targeting MRI patient experience, a study-specific questionnaire had to be developed. Although content and face validity were established through expert panel review and pilot pre-testing (n = 25), the instrument lacks formal psychometric cross-validation across diverse clinical cohorts. Future research should focus on validating standardized scales for public diagnostic imaging settings. Patient satisfaction was assessed using a self-report questionnaire; therefore, the findings may have been affected by respondent bias, current emotional state, or the immediate experience of the examination. Although the model demonstrated meaningful explanatory power, the remaining variance indicates that additional factors such as prior MRI experience, predisposition to claustrophobia, clinical diagnosis, examination duration, or previous experiences with the healthcare system may also influence satisfaction. Furthermore, the study was conducted within a specific institutional setting; therefore, the generalizability of the findings to other levels of care or radiology departments with differing organizational structures should be interpreted with caution.

An additional limitation of this study is that patient-perceived staff competence was assessed using a single-item measure. Although this item captured patients’ overall perceptions of staff competence, it could not fully reflect the multidimensional nature of professional competence, which encompasses technical knowledge, clinical skills, communication abilities, and patient-centered care. Furthermore, the study did not include an objective assessment of the clinical or technical competence of healthcare professionals. Future research should employ validated multi-item instruments to provide a more comprehensive evaluation of perceived competence and distinguish between the different professional groups involved in the MRI care pathway.

Practical Implications

To move beyond generic recommendations, our empirical findings point to three specific, actionable operational strategies tailored to public MRI departments:

1. Standardized Pre-Scan Micro-Briefings: To address spatial isolation and claustrophobic anxiety, radiographers should implement a standardized 45-to-60-s verbal “micro-briefing” prior to table positioning. This protocol must explicitly cover sequence duration, acoustic expectations, and immediate panic-button responsiveness, which directly strengthens diagnostic confidence (β = 0.208) and perceived communication safety.
2. Targeted Soft-Skills Training for Radiographers: Given that staff competence (β = 0.349) is the strongest predictor of overall satisfaction, hospital management should reallocate continuing education budgets toward accredited interpersonal communication and anxiety de-escalation modules for technical staff.
3. Proactive Delay Communication Protocols: Since process-level communication buffers structural waiting time friction, administrative staff should adopt an active update protocol—verbally informing waiting patients every 15–20 min about schedule shifts. Managing expectations in real time preserves institutional trust without requiring costly physical waiting room renovations.

## 5. Conclusions

Our findings indicate that patient satisfaction with MRI examinations is not determined solely by technological or organizational factors but is strongly shaped by human interactions and the perceptual environment. Based on our empirical model, we propose three concrete, actionable clinical interventions for radiology departments to enhance patient experience and operational quality:

1. Structured Pre-Examination Dialogue: Communication should extend beyond standardized safety checklists. Radiographers should explicitly communicate the diagnostic purpose of the examination, expected duration, and acoustic features. Clear framing of the scan’s diagnostic efficacy directly builds clinical trust (β = 0.208), reinforcing patient willingness to tolerate physical discomfort.
2. Active In-Scan Communication Protocols: To counteract the distressing negative effect of perceived isolation (β = −0.131), radiology staff should implement structured voice-intercom check-ins between scan sequences (e.g., “We are halfway through; the next sequence will last 3 min and will sound like a rapid tapping”). Brief, reassuring verbal feedback bridges sensory deprivation and restores the patient’s sense of control.
3. Standardized Sensory and Environmental Reassurance: Environmental mitigation measures should be embedded into standard clinical workflows rather than treated as optional amenities. The routine provision of mirror glasses (enabling outside-bore visibility), active noise-canceling headphones with music, and explicit confirmation of the squeeze-bulb call button responsiveness provide tangible psychological security during spatial confinement.

## Figures and Tables

**Table 1:** Summary table of descriptive statistics.

	Mean ± Standard Deviation	Median	Minimum–Maximum	Number of Respondents
Overall satisfaction with the MRI examination (1–4)	3.76 ± 0.473	4	1–4	699
Access to MRI examination (days)	51.71 ± 53.7	31	0–365	627
Information provided (1–4)	3.58 ± 0.640	2	1–4	723
Disturbance caused by beeping sounds (1–4)	2.10 ± 0.944	2	1–4	711
Being alone during the examination (1–4)	1.64 ± 0.845	1	1–4	717
Professional competence of staff (1–4)	3.87 ± 0.406	4	1–4	706
Satisfaction with hygiene (1–4)	3.77 ± 0.474	4	1–4	710
Confidence in the effectiveness of the examination (1–4)	3.66 ± 0.517	4	1–4	719
Confidence in the safety of the examination (1–4)	3.67 ± 0.537	4	1–4	716

**Table 2:** Hierarchical multivariate regression model of factors explaining satisfaction with MRI examinations. Note: β: standardized regression coefficient. * *p* < 0.05. ** *p* < 0.001.

	Model 1 (β)	Model 2 (β)	Model 3 (β)	Model 4 (β)	Model 5 (β)	Model 6 (β)
Competence of healthcare professionals	0.591 **	0.482 **	0.469 **	0.375 **	0.358 **	0.349 **
R^2^ (%)	34.9%					
F (*p*)	310.96 (<0.001)					
Confidence in the effectiveness of the examination		0.311 **	0.296 **	0.270 **	0.223 **	0.208 **
R^2^ (%)		43.4%				
F (*p*)		221.91 (<0.001)				
Disruptive effects of being alone			−0.166 **	−0.148 **	−0.140 **	−0.131 **
R^2^ (%)			46.1%			
F (*p*)			164.46 (<0.001)			
Hygiene				0.160 **	0.146 **	0.128 **
R^2^ (%)				47.6%		
F (*p*)				131.15 (<0.001)		
Confidence in the safety of the examination					0.101 **	0.100 **
R^2^ (%)					48.2%	
F (*p*)					107.16 (<0.001)	
Information provided						0.082 *
R^2^ (%)						48.8%
F (*p*)						91.07 (<0.001)

**Table 3:** Collinearity statistics of the hierarchical multivariate regression model.

Variables	Tolerance	VIF
Professional competence of staff	0.597	1.676
Confidence in the effectiveness of the examination	0.621	1.610
Being alone during the examination	0.920	1.087
Satisfaction with hygiene	0.571	1.751
Confidence in the safety of the examination	0.574	1.743
Information provided	0.793	1.261

## Data Availability

The datasets generated and analyzed during the current study are available from the corresponding author upon reasonable request. The data are not publicly available due to ethical reasons.
